# Mantis Shrimp Locomotion: Coordination and Variation of Hybrid Metachronal Swimming

**DOI:** 10.1093/iob/obad019

**Published:** 2023-06-27

**Authors:** S E Hanson, W J Ray, A Santhanakrishnan, S N Patek

**Affiliations:** Department of Biology, Duke University, Durham, NC 27708, USA; Department of Biology, Duke University, Durham, NC 27708, USA; School of Mechanical and Aerospace Engineering, Oklahoma State University, Stillwater, OK 74078, USA; Department of Biology, Duke University, Durham, NC 27708, USA

## Abstract

Across countless marine invertebrates, coordination of closely spaced swimming appendages is key to producing diverse locomotory behaviors. Using a widespread mechanism termed hybrid metachronal propulsion, mantis shrimp swim by moving five paddle-like pleopods along their abdomen in a posterior to anterior sequence during the power stroke and a near-synchronous motion during the recovery stroke. Despite the ubiquity of this mechanism, it is not clear how hybrid metachronal swimmers coordinate and modify individual appendage movements to achieve a range of swimming capabilities. Using high-speed imaging, we measured pleopod kinematics of mantis shrimp (*Neogonodactylus bredini*), while they performed two swimming behaviors: burst swimming and taking off from the substrate. By tracking each of the five pleopods, we tested how stroke kinematics vary across swimming speeds and the two swimming behaviors. We found that mantis shrimp achieve faster swimming speeds through a combination of higher beat frequencies, smaller stroke durations, and partially via larger stroke angles. The five pleopods exhibit non-uniform kinematics that contribute to the coordination and forward propulsion of the whole system. Micro-hook structures (retinacula) connect each of the five pleopod pairs and differ in their attachment across pleopods—possibly contributing to passive kinematic control. We compare our findings in *N. bredini* to previous studies to identify commonalities across hybrid metachronal swimmers at high Reynolds numbers and centimeter scales. Through our large experimental dataset and by tracking each pleopod's movements, our study reveals key parameters by which mantis shrimp adjust and control their swimming, yielding diverse locomotor abilities.

## Introduction

**Fig. 1:**
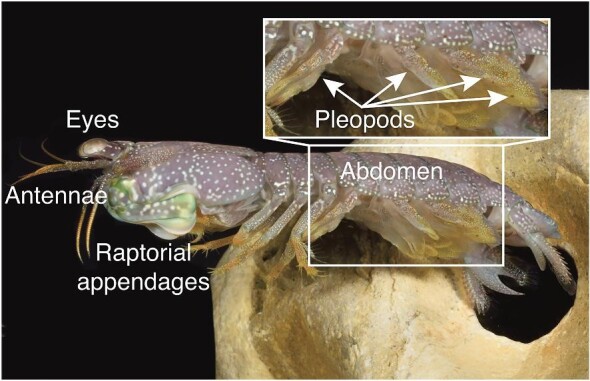
Mantis shrimp use abdominal appendages, called pleopods, for swimming and breathing. They swim using hybrid metachrony, consisting of an asynchronous, metachronal power stroke and near-synchronous recovery stroke of their five pleopods. The zoomed-in region of the abdomen illustrates pleopods P1–P4 (anterior to posterior; left to right in this image); P5 is not visible. *Neogonodactylus bredini* is shown here; photo courtesy of Roy Caldwell.

**Fig. 2:**
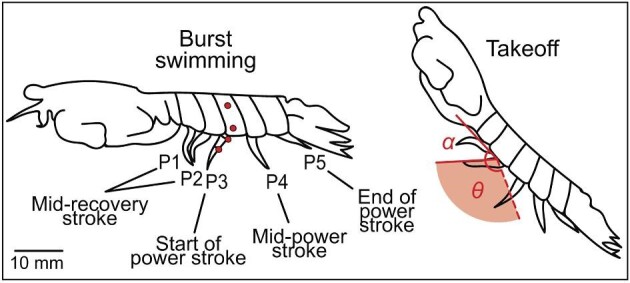
Hybrid metachrony consists of a metachronal power stroke and near-synchronous recovery stroke. Power and recovery stroke phases are illustrated for each pleopod in a lateral view of a hybrid metachronal sequence (lateral view; anterior to left). Pleopods are labeled P1–P5, numbered from anterior to posterior. We compare stroke kinematics of two locomotor behaviors, burst swimming (left) and takeoff (right). Both illustrations are traced from high-speed videos; walking and feeding appendages are excluded from the illustrations. Red points represent the placement of landmarks used for digitizing kinematics, located on two joints on the pleopod and two distinct, natural markings on the same abdominal somite segment as the focal pleopod (in this case, P3). α is the angle between the pleopod and the long axis of the body. Stroke angle (θ) is the angle the appendage sweeps during the power stroke, from start of the power stroke (solid red line extending from P2) to the end of the power stroke (dashed red line). The total stroke angle is shown in red shading.

Swimming crustaceans have different locomotory needs depending upon their environment and ecological niche (Hessler 1981; Vogel 2008; Biewener and Patek 2018). Some require the ability to hover and migrate long distances, while others need short bursts of speed or the maneuverability to navigate within burrows. Across these diverse locomotory needs, drag-based propulsion by the rowing of multiple swimming appendages (called pleopods) is a common form of locomotion amongst freely swimming aquatic crustaceans (Sleigh and Barlow 1980; Vogel 2008; Alben et al. 2010). Closely spaced pleopods are coordinated to rhythmically paddle in an adlocomotory sequence, such that the posterior pleopod initiates the propulsive stroke and, after some time delay, is followed by the neighboring anterior pleopod (Figs 1 and 2). This sequential motion, known as metachronal rowing, results in the appearance of a wave progressing in the direction of the animal's movement (Knight-Jones and Macfadyen 1959; Sleigh and Barlow 1980).

Crustaceans exhibit two types of metachronal rowing—complete metachrony and hybrid metachrony—defined by differences in the timing of stroke phases. Stroke phases are comprised of the power stroke (the propulsive motion) and the recovery stroke (the re-positioning of appendages for propulsion). Complete metachrony is defined by a metachronal motion during both the power and the recovery stroke (e.g., Alben et al. 2010; Murphy et al. 2011; Ford et al. 2019; Ford and Santhanakrishnan 2021a; Ford and Santhanakrishnan 2021b; Ruszczyk et al. 2022). Conversely, hybrid metachrony is defined by a metachronal motion during the power stroke but a unified, near-synchronous motion during the recovery stroke (Alexander 1988; van Duren and Videler 2003; Kiørboe et al. 2010; Campos et al. 2012; Heimbichner Goebel et al. 2020; Ford et al. 2021; Garayev and Murphy 2021).

Hybrid metachrony, complete metachrony, and other metachronal mechanisms are used in diverse ways across crustacean sizes and taxonomy (Byron et al. 2021). For instance, endurance swimmers like Antarctic krill—known to vertically migrate hundreds of meters per day—use complete metachronal swimming (Alben et al. 2010; Murphy et al. 2011). Jet-assisted walking in lobsters utilizes a metachronal power stroke and phase-shifted grouping during the recovery stroke (Lim and DeMont 2009). Some mysid shrimp beat their left and right pleopods independently of each other in a dual-phase metachronal sequence (Ruszczyk et al. 2021). Hybrid metachronal swimmers span a staggering range of sizes, from barnacle larvae (*Re* = 1, body length = 476 μm; Wong et al. 2020; Lamont and Emlet 2021) to large deep-sea amphipods (body length < 12 cm; Boudrias 2002). Hybrid metachrony has been described in copepods (Alcaraz and Strickler 1988; van Duren and Videler 2003; Kiørboe et al. 2010), amphipods (Boudrias 2002), isopods (Alexander 1988), and stomatopods (Campos et al. 2012; Garayev and Murphy 2021; Ford et al. 2021). Animals that use hybrid metachronal propulsion are capable of large accelerations and agile maneuvers (Ford et al. 2021).

Regardless of the type of metachrony used, swimming capabilities are governed by morphological and kinematic parameters of the swimming appendages, including beat frequency, stroke duration, or appendage angle (α). Stroke kinematics can be modified to achieve varying swimming speeds (Alexander 1988; Murphy et al. 2011; Ford et al. 2021) as well as distinct swimming modes, such as fast-forward, hovering, or upside-down swimming in krill (Murphy et al. 2011; Ruszczyk et al. 2022). Great kinematic variation of these parameters is present across crustaceans (Ruszczyk et al. 2022).

While metachronal mechanisms and stroke kinematics are central to aquatic locomotion in numerous animals, how organisms modify kinematics within a hybrid metachronal system is also important for achieving different locomotor behaviors and swimming speeds. Mantis shrimp (Stomatopoda) are well-suited for studies of hybrid metachrony across different swimming behaviors and various speeds (Campos et al. 2012; Ford et al. 2021; Garayev and Murphy 2021). Mantis shrimp are not only highly maneuverable swimmers capable of fast bursts, but they also use their five pairs of pleopods for crawling, digging, and respiration. Unlike other studies of hybrid metachronal swimmers that operate in low or intermediate Reynolds numbers (*Re*) (e.g., copepods; van Duren and Videler 2003), mantis shrimp operate at high *Re* in the inertial realm (*Re *> 10^3^; Garayev and Murphy 2021; see Results).

Here, we address how stroke kinematics within a hybrid metachronal system vary within and across two swimming behaviors. We ask the following two questions: (1) How do mantis shrimp achieve hybrid metachrony through spatial and temporal coordination of their pleopod movements? And (2) How does variation in stroke kinematics enable different swimming behaviors and speeds? In the spatial realm, we measure how a pleopod's range of motion varies across the five pleopod pairs (via stroke angle and α). In the temporal realm, we measure stroke duration, beat frequency, and the time delay between consecutive pleopod strokes (i.e., phase lag). Given our current understanding of hybrid metachrony from robotic modeling, mantis shrimp should be able to increase swimming speed by increasing stroke angle, while maintaining near constant timing of pleopod motion (Ford et al. 2021). In other words, swimming bouts with larger average stroke angles should positively correlate with greater swimming speeds. Because larger animals swim faster (Lenz et al. 2004; Heimbichner Goebel et al. 2020; Svetlichny et al. 2020), we incorporate body size as an additional predictor for this relationship. Previous studies have explored kinematics and speed in mantis shrimp (Campos et al. 2012; Ford et al. 2021; Garayev and Murphy 2021). However, to our knowledge, this is the first analysis of the mechanisms by which hybrid metachronal propulsion can achieve different swimming behaviors. In addition to our kinematic study, we describe small, intricate hooks (termed retinacula) that connect left and right pleopod pairs (Bouchon and Chaigneau 1991) and compare our kinematic findings to other mantis shrimp species that operate at similar *Re*s.

## Methods

### Animals

Adult mantis shrimp (Crustacea: Stomatopoda: Gonodactylidae: *N. bredini*) were collected at the Galeta Marine Station, Smithsonian Tropical Research Institute, Panama (Permit # SC/A-6-19). They were housed individually in artificial saltwater tanks at Duke University (44 liter tanks, 12 h:12 h light: dark cycle; 27–28°C, salinity 32–36 parts per thousand) and fed three times per week with frozen and fresh seafood. Experiments were performed on 10 animals in their individual home tanks.

### Morphology

To examine pleopod morphology, mantis shrimp (not used in the locomotion study) were euthanized in a small container with sea water placed in a freezer. Mantis shrimp were then placed in a petri dish filled with sea water and examined under a stereomicroscope (model 165M FC stereomicroscope, Leica Microsystems Inc., Buffalo Grove, IL, USA). Pleopods were removed from the abdomen and images were acquired using a camera mounted on the stereomicroscope (2560 × 1920 pixel resolution; DFC 450 C camera; model 165M FC microscope; Leica Microsystems Inc., Buffalo Grove, IL, USA).

For mantis shrimp used in the locomotion study, body length–the distance from tip of the rostrum to the middle fork of the telson–was measured using calipers (0.01 mm resolution, accuracy +/0.02 mm, Fine Science Tools Foster City, CA, USA). Body length was measured three times and then averaged to reduce errors due to animal positioning. Animals were also weighed using a microbalance (XPE56 Mettler Toledo, 1 μg resolution, Pleasant Prairie, WI, USA). Wet body mass was recorded three times and then averaged for each individual.

### Burst swimming and takeoff experiments

Burst swimming and takeoff behaviors (Fig. 2; Supplementary Videos S1, S2) were filmed in each animal's home tank. We elicited burst swimming by enticing mantis shrimp to pursue bait (held in long forceps), such that they swam laterally within the focal plane of the camera and in a linear path. Burst swimming occurs when animals accelerate while already in motion with a nearly horizontal body angle. We elicited takeoff behavior by baiting the mantis shrimp to crawl along the substrate until in the focal plane and then raising the bait up into the water column. We define takeoff as the first complete swimming cycle in which none of the pleopods are in contact with the substrate from the beginning of the recovery stroke of P5. We excluded videos with excessive roll or yaw of the body. We defined roll or yaw as occurring when any of the following parts of the animal were visible: ventral side of the animal, uropod and tip of the forked telson that were on the side of the animal furthest from the camera, and the protopodite of any pleopod pair furthest from the camera. In all, 170 videos with at least one complete stroke cycle for an entire set of pleopods were collected, of which 33 burst swimming and 15 takeoff videos were selected for further analysis. Fewer takeoff videos were collected because of our strict selection criteria and loss of animals in between the burst swimming and takeoff swimming experiments. Prior to the experiments, mantis shrimp were acclimated to pursue their food in the presence of the lights used for high-speed imaging (75 W LED, Varsa, Nila, Inc., Altadena, CA, USA).

### Stroke kinematics

In order to compare spatial and temporal kinematics of pleopods across burst swimming and takeoff behaviors, we measured six stroke variables: stroke angle, minimum and maximum α, power stroke duration, recovery stroke duration, beat frequency, and phase lag (defined in Table 1). Pleopod pairs are identified as P1–P5, with the anterior-most pleopod defined as P1 and the posterior-most pleopod defined as P5 (Fig. 2).

**Table 1:** Definitions of variables used in this study.

Variable	Units	Definition
P1, P2, P3, P4, P5	–	Refers to the five pleopods numbered from anterior (P1) to posterior (P5)
Stroke angle	°	Pleopod excursion angle from start to end of the power stroke
Appendage angle (α)	°	Anterior-facing angle between first segment of a pleopod (protopodite) and the long axis of the body
Recovery stroke	s	Duration of the pleopod moving in the same direction of the animal's motion, nearly synchronously with other pleopods
Power stroke	s	Duration of the pleopod moving in the opposite direction of the animal's motion, metachronally or asynchronously to other pleopods
Complete cycle	s	Duration of the recovery and power stroke; can either refer to a cycle for one pleopod or a cycle for an entire set of pleopods (i.e., start of P5’s recovery to end of P1’s power stroke)
Beat frequency	Hz	Reciprocal of the complete cycle duration for each pleopod
Phase lag	%	Time between the start of consecutive power strokes by each pleopod divided by the duration of a complete cycle for an entire set of pleopods
Swimming speed	m s^−1^	Average body speed recorded during the complete stroke cycle of each pleopod
Normalized swimming speed	BL s^−1^	Swimming speed divided by animal's body length (m)
Body angle	°	Angle of the long axis of the body across several abdominal segments relative the substrate (Fig. S1)
Appendage spacing ratio	G/L	Average distance between pleopods (*G*) normalized by average pleopod length (*L*)

All measurements are briefly defined in Table 1. Specifically, we measured stroke angle (also known as stroke amplitude) and α using the first segment of the pleopod (protopodite) to determine the pleopod's position (Fig. 2). We measured the recovery stroke duration from one video frame before a pleopod appears to initiate movement in the same direction as the animal's motion to the video frame before a pleopod begins to move in the opposite direction as the animal's motion (filmed at 500 frames s^−1^). Similarly, the power stroke duration was measured from one video frame before the pleopod begins to move in the opposite direction of the animal's motion to the video frame before the pleopod appears to cease movement toward the body. Phase lag is the time elapsed between the start of sequential power strokes divided by the complete stroke cycle duration for all pleopods (i.e., start of P5’s recovery stroke to the end of P1’s power stroke), expressed as a percentage. We characterized phase lag as P5–P4, P4–P3, P3–P2, and P2–P1 (referred to as “within-cycle” phase lag). We did not measure “between-cycle” phase lag (P1–P5).

Kinematics were recorded for each individual pleopod and then averaged to determine the mean value for each complete swimming cycle.

### High-speed imaging and analysis

To measure the kinematic variables described above, we collected and analyzed high-speed videos. We recorded burst swimming and takeoff behaviors at 500 frames s^−1^ using high-speed imaging (lens: AF-S VR Micro-Nikkor 105mm f/2.8G 1:2.8 D). The high-speed video camera used to film alternated between two models (1024 × 1024 pixel resolution, 0.8606 ms shutter duration; FASTCAM SA-Z type 2100K-M-64GB and FASTCAM SA-X2 type 1000K-M4-64GB; Photron, San Diego, CA, USA). High-speed images were calibrated using a ruler placed in the frame of view and focal plane of the video camera. The ruler was calibrated to 0.01 mm using a stage micrometer under a stereo microscope (KR-814 (1×3), Klarmann Rulings, Inc., Litchfield, NH, USA, model 165M FC stereomicroscope and DFC 450 C camera 2560 × 1920 pixel resolution; Leica Microsystems Inc., Buffalo Grove, IL, USA). The calibration ruler and a zip-tie were positioned on the substrate within the camera's focal plane and videos were constrained to this focal plane. Image analyses and video selection were performed using ImageJ (version 2.0.0-rc-69/1.52p National Institutes of Health, USA).

For each pleopod, we digitized three frames: (1) the start of the recovery stroke, (2) the end of the recovery stroke (equivalent to the start of the power stroke), and (3) the end of the power stroke. The “rest” position of the pleopods occurs between the end of the power stroke and start of the recovery stroke; therefore, a single cycle begins with the recovery stroke, such that the sequence follows a recovery-power cycle, not a power-recovery cycle as may be found in other metachronal swimmers.

Pleopod length was measured from the high-speed videos at the start of the power stroke when the pleopod was fully extended. We measured along the pleopod's lateral edge (facing the high-speed camera) from the point at which the abdominal somite intersected the protopodite (base of pleopod) to the exopodite (tip of the pleopod), excluding the setae.

To calculate the stroke angle, we modified the four-point digitization method developed by Kagaya and Patek (2016), which calculates the angle between the intersection of two lines and does not require the specification of a pivot point. We used two digital landmarks on the pleopod and two on the body to calculate the change in the pleopod's angle across the three video frames (Fig. 2). The distal point on the pleopod was located on the exopodite–protopodite joint, and the proximal point on the pleopod was located on the joint of the protopodite and pleopod base. The two body landmarks were located on distinct natural markings on the same abdominal somite segment of the pleopod being analyzed (Fig. 2). To improve accuracy, natural markings that were farther apart on the corresponding abdominal somite were chosen over markings located close to each other. From these four digitized points, two lines were formed: one along the two pleopod points and one along the two body points. The latter served as a reference line used to account for the body's movement. We calculated the stroke angle using the difference in angles between the pleopod line and body line during the power stroke (Kagaya and Patek 2016).

We also calculated minimum and maximum α to identify the pleopod's position relative to the long axis of the body. First, we digitized a line parallel to the long axis of the animal's body in ImageJ (version 2.0.0-rc-69/1.52p National Institutes of Health, USA; Fig. S1). The long axis of the body was identified separately for each of the five abdominal segments specific to each pleopod (specifically when the pleopods were at the beginning of their power strokes), and the line was extended along the lip of the abdominal somite using the base of neighboring pleopods to best approximate the body's angle (Fig. S1). To approximate the body angle across the complete swimming cycle for an entire set of pleopods, the average was then taken across these five lines drawn along the body's long axis for each of the five pleopods at the same stage of their respective stroke cycles. Body angle is expressed as the angle of a left-facing animal relative to the substrate (or positive *x*-axis). To calculate α, we took the anterior-facing angle formed by the intersection of the pleopod line and the line along the long axis of the body. α was calculated at each of the three video frames for each pleopod using the body points as a reference for body movement. From the three measured angles, we calculated the minimum and maximum α, representing static pleopod extrema. Finally, to verify our stroke angle calculations above, we calculated stroke angle using an alternative method by subtracting minimum α from maximum α. Supplementary Materials (Tables S1 and S2) include a comparison of these two methods for calculating stroke angle. For the remainder of the paper, we report values of stroke angle from the four-point digitization method and α angle from the digitization of the long axis of the body.

In addition to stroke kinematics, we measured the body's speed across the duration of each pleopod's recovery-power stroke cycle. The speed of the animal's body was tracked using a digital landmark on the abdominal somite of the pleopod being digitized. The average body speed across a complete stroke cycle for an entire set of pleopods (i.e., start of P5’s recovery stroke to end of P1’s power stroke) was then calculated by averaging these five pleopod-specific body speeds.

Finally, we calculated *Re*, a dimensionless parameter calculated as the ratio of inertial to viscous fluid forces (Vogel 1994). *Re* is defined as *UL/*ν, where *U* is the characteristic velocity, *L* is the characteristic length scale, and ν is the kinematic viscosity of the fluid (Vogel 1994). We estimated peak *Re* of the mantis shrimp's body by using maximum swimming speed and body length. We also estimated the peak pleopod *Re* using the maximum speed of the pleopods and the average pleopod length across the five appendages. We used a reference value for the kinematic viscosity of seawater (ν = 0.851 × 10^−6^ m^2^ s^−1^) that approximates the tanks’ conditions (35‰ at 30°C; Vogel 1994).

To calculate and analyze the kinematic data, the *x* and *y* coordinates for the digital landmark points were processed in R (version 4.2.2 GUI 1.79 High Sierra build (8160), R studio version 2023.03.0 + 386; R Core Team 2022). Data and R code are made available at Dryad (https://doi.org/10.5061/dryad.x95x69pq6; Hanson et al. 2023).

### Statistical analysis

We generated linear mixed models (LMMs) using the lme4 R package (Bates 2015) to test which parameters: (1) significantly differed between the two behaviors, (2) best predicted swimming speed, and (3) significantly differed across the five pleopods. For the first two objectives, data was averaged across the five pleopods, such that one data point represented one swimming cycle, with multiple swimming cycles from each animal, including cycles from both behaviors (*n* = 48 cycles). We first tested the distribution of the data for normality and then tested whether individual ID significantly explained variation in kinematic parameters. Given that we found a statistically significant influence of ID on the parameter relationships, we accounted for ID as a random effect in all subsequent LMMs.

To test which parameters significantly differed between the two behaviors, we generated separate models each with behavior as the fixed effect and the response variable as either swimming speed, body length, recovery stroke duration, power stroke duration, beat frequency, stroke angle, or body angle (seven models in total). All models were allowed to vary by random intercepts. To assess model fit, we calculated the difference between the Akaike Information Criterion (AIC) score of the model and the AIC score of a respective null model (ΔAIC = AIC_parameter model_—AIC_null_). For all LMMs in each of the three objectives, a ΔAIC score < -2 indicated a significant model fit (Raferty 1995; Burnham and Anderson 2004). Given that each ΔAIC was computed relative to each model's respective null, ΔAIC was not used to rank or compare fit across models—only to indicate model significance (ΔAIC scores reported in Table S3). Raw AIC scores and LMMs are available in the R code at Dryad (https://doi.org/10.5061/dryad.x95x69pq6; Hanson et al. 2023).

To evaluate the effect of morphological and kinematic parameters on swimming speed, we generated two sets of LMMs. In the first set, we ran separate models with swimming speed as the response variable and behavior, body length, recovery stroke duration, power stroke duration, beat frequency, stroke angle, phase lag, and body angle as the fixed effect in each model (eight models in total). Individual ID was kept as a random effect in all models. Here, the AIC score of each model was compared to only one null model (with swimming speed as the response variable, no fixed effect, and individual ID as the random effect). Therefore, ΔAIC scores were used to compare fit across models, such that the lowest ΔAIC value indicated best fit and best predictor of swimming speed (ΔAIC scores reported in Table S4).

Given that behavior was the most significant predictor of swimming speed out of the models tested above (see Results), we then included behavior as an additional random effect in the second set of LMMs (structured as (1 | ID) + (1 | behavior)). Again, we generated separate models with swimming speed as the response variable and body length, recovery stroke duration, power stroke duration, beat frequency, stroke angle, phase lag, and body angle as the fixed effect in each model (seven models in total). To evaluate fit, we generated a null model with the same crossed random effects and calculated ΔAIC against the null. ΔAIC scores were used to compare best fit across models, such that the lowest ΔAIC value indicated best fit and best predictor of swimming speed (ΔAIC scores reported Table S5).

Finally, we generated LMMs to test which parameters significantly differed across the five pleopods using stroke kinematics specific to each pleopod (*n *= 240 pleopods across 48 swimming cycles). Given that behavior significantly explained variation in pleopod stroke kinematics, we included behavior as a crossed random effect along with individual ID. We generated independent models with pleopod number (P1–P5) as the fixed effect and pleopod length, recovery stroke duration, power stroke duration, beat frequency, stroke angle, minimum α, and maximum α as the response variable in each model (seven models in total). We then compared each model to its respective null (with the respective response variable, no fixed effect, and both individual ID and behavior as random effects). To evaluate model significance, we calculated ΔAIC scores respective to the null for each model, such that ΔAIC was not used to rank or compare fit across models—only to indicate model significance (ΔAIC scores reported in Table S6).

To calculate digitizing error, we digitized one pleopod in one video 40 times. We report a standard error of the mean of 2.05% for pleopod length, 0.28% for swimming speed, 2.90% for stroke angle (four-point method modified from Kagaya and Patek (2016)), and 2.78% for stroke angle (α subtraction method). If either notch on the posterior side of the two pleopod joints was not sufficiently visible to place a landmark, then we used the anterior side of the joints (or a combination of posterior and anterior side of the two pleopod joints). There are four possible combinations of anterior–posterior landmark placements on the two pleopod joints. To test whether this affected the calculation of stroke angle, we digitized a video 10 times for each of the four combinations, resulting in 40 total digitizations. We found no significant difference in digitizing error across the four different combinations.

### Comparative kinematics across mantis shrimp species

To compare stroke kinematics and general morphology across other hybrid metachronal swimmers that operate at similar *Re*, we compiled data from published studies of other mantis shrimp species: *Odontodactylus havanensis* from Campos et al. (2012), *Odontodactylus scyllarus* from Garayev and Murphy (2021), and *N. bredini*, the same species used in this study, from Ford et al. (2021) (compiled in Table 2). For *O. havanensis*, we used mean values from published data to represent one trial reported in Table 2 of this study (Campos et al. 2012), and these data were then supplemented with unpublished kinematics extracted by Ruszczyk et al. (2022) for the remaining three trials. For *O. scyllarus*, we excluded data that were not measured in the sagittal plane from Garayev and Murphy (2021), and, consequently, excluded larger reported swimming speeds for *O. scyllarus* (0.57 m s^−1^ and 1.9 m s^−1^). In addition, Garayev and Murphy (2021) only document stroke angle for P5 and minimum and maximum α for one representative observation in the study. We reported these measurements in Table 2. Finally, for *N. bredini*, data were compiled from the Supplementary Materials provided by Ford et al. (2021), which were reported as median values instead of means. Given that this current study uses the same species and animals of a similar size to those used by Ford et al. (2021), we report the same *G/L* documented in Ford et al. (2021) for animals in this study in Table 2. Note that the videos used in Ford et al. (2021) were collected in the same facilities and with the same methodological criteria as animals in our study, but stroke kinematics were extracted independently.

**Table 2:** Comparative stroke kinematics across mantis shrimp species. Data from the present study and previous studies were compiled to compare stroke kinematics across closely-related taxa. Values are reported as the overall mean followed by the range in parentheses, except for stroke angle in Ford et al. (2021) which is reported as median values (see Methods for details).

Species	*n*	Trials	Behavior	Body length (mm)	G/L	Minimum α (°)	Maximum α (°)	Stroke angle (°)	Beat frequency (Hz)	Within-cycle phase lag (%)	Swimming speed (m s^-1^)	Normalized swimming speed (BL s^-1^)	Reference
*N. bredini*	10	33	Burst swimming	50.99 (43.77–56.22)	0.56	44 (34–66)	155 (145–165)	108 (89–118)	9.8 (6.1–13.6)	9.4 (8.5–10.7)	0.28 (0.19–0.40)	5.40 (3.75–7.04)	This study
*N. bredini*	4	13	Burst swimming	54.6 (50.6–57.9)	0.56	–	–	117 (101–133)	8.5 (6.9–9.6)	15.0 (9–25)	0.29 (0.19–0.42)	5.2 (3.3–8.0)	Ford *et al.* 2021
*O. scyllarus*	1	2	Escape swimming	114.00	0.34	(27–40)	(126–143)	110 (106–114)	4.2 (3.6–4.8)	15 (14.5–15.5)	0.30 (0.20–0.39)	2.6 (1.8–3.4)	Garayev and Murphy 2021
*O. havanensis*	4	4	Escape swimming	54 (50–63)	0.56	24 (19–28)	169 (166–174)	138 (124–154)	16.8 (13.7–19.3)	9.1 (7.0–10.3)	1.31 (1.09–1.47)	24.1 (21.0–28.7)	Campos *et al.* 2012 and Ruszczyk *et al.* 2022

*n* is number of animals– indicates that data was either not reported or not extractable.

## Results

Six kinematic parameters defined the spatial and temporal characteristics of hybrid metachronal swimming: stroke angle, α, power stroke duration, recovery stroke duration, beat frequency, and phase lag. Variation of these kinematics occurred within and across individuals and two swimming behaviors (burst swimming: Table 3, takeoff: Table 4), across the five pleopods (Table 5), and across consecutive pleopod strokes (in the case of phase lag; Table S7).

**Table 3:** Kinematics of burst swimming behavior. A total of 33 complete swimming cycles (recovery stroke + power stroke) were analyzed across ten individuals. Body length and body mass were averaged across three measurements. Bottom row contains overall mean ± standard deviation of means, followed by the range in parentheses. Stroke angle is reported from the four-point digitization method.

ID	Trials	Body length (mm)	Body mass (g)	Swimming speed (×10^–1^ m s^-1^)	Normalized swimming speed (BL s^-1^)	Body angle (°)	Minimum α (°)	Maximum α (°)	Stroke angle (°)	Power stroke duration (×10^–2^ s)	Recovery stroke duration (×10^–2^ s)	Beat frequency (Hz)	Phase Lag (%)
A	1	56.22	4.06	4.00	7.04	176	37	157	118	3.5	5.8	11.0	9.9
B	1	53.48	3.34	3.32	6.20	183	34	162	117	3.6	5.0	11.8	10.7
C	6	48.90	2.84	2.64 (2.15–3.38)	5.40 (4.39–6.92)	178 (164–193)	42 (37–50)	161 (153–169)	117 (113–125)	4.1 (2.8–5.4)	8.9 (4.4–14.9)	8.6 (4.9–14.0)	8.7 (7.0–11.1)
D	2	53.06	3.80	3.00 (2.33–3.68)	5.66 (4.40–6.93)	180 (173–187)	66 (60–71)	154 (147–162)	89 (87–90)	3.3 (2.7–3.9)	4.4 (3.4–5.5)	13.6 (10.6–16.6)	8.8 (8.0–9.6)
E	5	48.04	2.55	2.84 (2.70–2.93)	5.92 (5.62–6.11)	183 (169–190)	44 (34–63)	165 (154–175)	116 (102–129)	3.9 (3.3–4.4)	9.4 (8.2–10.6)	7.7 (6.7–8.7)	8.5 (7.6–9.4)
F	3	50.44	3.40	2.46 (2.22–2.72)	4.87 (4.40–5.40)	174 (161–190)	52 (46–55)	158 (151–170)	104 (90–116)	4.0 (3.2–4.5)	7.7 (5.1–9.9)	9.2 (7.1–12.0)	9.3 (3.6–13.7)
G	4	52.63	3.44	3.09 (2.79–3.48)	5.87 (5.29–6.61)	164 (150–178)	38 (36–40)	153 (149–159)	114 (106–119)	3.0 (2.7–3.1)	5.5 (4.7–6.6)	12.1 (10.4–13.5)	10.3 (9.1–12.3)
H	2	52.96	3.73	1.99 (1.88–2.09)	3.75 (3.55–3.95)	180 (174–185)	42 (42–42)	145 (144–145)	99 (98–101)	5.1 (5.0–5.2)	11.4 (10.5–12.4)	6.1 (5.7–6.4)	8.8 (8.3–9.2)
I	3	43.77	4.62	1.94 (1.85–2.03)	4.44 (4.23–4.63)	179 (165–203)	46 (43–49)	148 (137–157)	99 (90–109)	3.7 (3.3–4.2)	7.2 (4.6–9.6)	9.8 (7.5–12.7)	10.1 (9.9–10.3)
J	6	50.35	3.34	2.43 (2.02–3.03)	4.82 (4.00–6.02)	180 (165–191)	41 (32–49)	150 (141–163)	108 (97–117)	4.0 (3.4–5.2)	8.9 (6.6–14.2)	8.2 (5.2–9.8)	8.9 (8.3–9.6)
Overall mean ± standard deviation (min–max)		50.99 ± 3.50 (43.77–56.22)	3.51 ± 0.59 (2.55–4.62)	2.77 ± 0.62 (1.94–3.96)	5.40 ± 0.95 (3.75–7.04)	178 ± 6 (164–183)	44 ± 9 (34–66)	155 ± 6 (145–165)	108 ± 10 (89–118)	3.8 ± 0.6 (3.0–5.1)	7.4 ± 2.3 (4.4–11.4)	9.8 ± 2.3 (6.1–13.6)	9.4 ± 0.8 (8.5–10.7)

**Table 4:** Kinematics of takeoff behavior. A total of 15 complete swimming cycles (recovery stroke + power stroke) were analyzed across six individuals. Values are reported as means and ranges. Bottom row contains overall mean ± standard deviation of means followed by the range in parentheses. Stroke angle is reported from the four-point digitization method.

ID	Trials	Body length (mm)	Body mass (g)	Swimming speed (×10^–1^ m s^-1^)	Normalized swimming speed (BL s^-1^)	Body angle (°)	Minimum α (°)	Maximum α (°)	Stroke angle (°)	Power stroke duration (×10^–2^ s)	Recovery stroke duration (×10^–2^ s)	Beat frequency (Hz)	Phase lag (%)
C	2	48.90	2.84	1.68 (1.04–2.32)	3.44 (2.13–4.75)	160 (153–167)	65 (50–80)	169 (164–173)	101 (88–114)	4.4 (4.1–4.7)	7.6 (7.3–8.0)	8.4 (8.3–8.5)	8.3 (7.8–8.8)
D	1	53.06	3.80	0.96	1.81	208	47	152	105	4.1	14.8	5.7	5.7
E	4	48.04	2.55	1.49 (0.71–1.97)	3.09 (1.47–4.10)	164 (158–166)	49 (42–67)	152 (143–165)	99 (91–104)	3.7 (2.8–4.8)	6.3 (4.5–7.7)	10.6 (8.0–13.8)	10.6 (9.2–11.6)
F	1	50.44	3.40	2.00	3.97	123	51	150	88	4.0	5.7	10.4	11.2
G	3	52.63	3.44	1.89 (1.79–2.03)	3.59 (3.40–3.86)	150 (136–159)	49 (45–56)	151 (147–158)	100 (98–102)	3.5 (2.7–4.2)	6.1 (4.3–8.1)	11.0 (8.3–14.3)	10.0 (8.9–11.7)
I	4	43.77	4.62	1.01 (0.62–1.42)	2.31 (1.40–3.23)	176 (152–225)	51 (45–63)	149 (140–158)	92 (74–110)	4.3 (3.8–5.2)	9.9 (7.2–15.8)	7.6 (4.8–9.4)	9.6 (7.1–12.2)
Overall mean ± standard deviation (min–max)		49.48 ± 3.43 (43.77–53.06)	3.44 ± 0.73 (2.55–4.62)	1.51 ± 0.44 (0.96–2.00)	3.04 ± 0.82 (1.81–3.97)	163 ± 28 (123–208)	52 ± 6 (47–65)	154 ± 7 (149–169)	98 ± 6 (88–105)	4.0 ± 0.3 (3.5–4.4)	8.4 ± 3.5 (5.7–14.8)	9.0 ± 2.1 (5.7–11.0)	9.2 ± 2.0 (5.7–11.2)

**Table 5:** Pleopod stroke kinematics across burst swimming and takeoff behaviors. Values are reported as means and ranges. Bottom row contains overall mean ± standard deviation of means followed by the range in parentheses. Stroke angle is reported from the four-point digitization method.

		Burst swimming behavior					Takeoff behavior				
Pleopod	Pleopod length (mm)	Swimming speed (×10^–1^ m s^-1^)	Minimum α (°)	Maximum α (°)	Stroke angle (°)	Beat frequency (Hz)	Swimming speed (×10^–1^ m s^-1^)	Minimum α (°)	Maximum α (°)	Stroke angle (°)	Beat frequency (Hz)
P1	7.71 (5.75–10.47)	2.86 (1.99–4.37)	35 (14–118)	147 (107–208)	109 (79–139)	8.9 (4.5–17.2)	1.64 (0.77–2.71)	36 (21–50)	141 (124–164)	100 (69–118)	9.0 (4.5–14.3)
P2	7.46 (6.24–9.78)	2.76 (1.94–4.26)	35 (17–59)	152 (130–172)	114 (66–144)	8.9 (4.7–16.7)	1.55 (0.68–2.54)	45 (28–90)	147 (127–167)	100 (65–122)	8.8 (4.5–13.9)
P3	7.50 (4.90–10.17)	2.64 (1.82–3.69)	42 (30–78)	161 (136–183)	117 (91–147)	8.8 (4.7–15.2)	1.49 (0.54–2.32)	50 (30–81)	155 (127–178)	101 (84–122)	9.2 (5.0–14.3)
P4	6.92 (5.31–8.93)	2.55 (1.70–4.00)	49 (34–92)	161 (147–184)	110 (79–128)	9.3 (5.1–16.7)	1.38 (0.56–2.10)	59 (45–88)	163 (145–195)	98 (70–121)	9.3 (4.7–14.7)
P5	6.28 (5.23–8.61)	2.45 (1.65–3.57)	59 (36–88)	157 (137–178)	97 (71–119)	10.5 (5.6–18.5)	1.27 (0.53–2.21)	69 (43–135)	159 (146–189)	87 (44–106)	10.0 (4.6–15.2)
Overall mean ± standard deviation (min–max)	7.17 ± 0.58 (6.28–7.71)	2.65 ± 0.16 (2.45–2.86)	44 ± 10 (35–59)	156 ± 6 (147–161)	109 ± 8 (97–117)	9.3 ± 0.7 (8.8–10.5)	1.47 ± 0.14 (1.27–1.64)	52 ± 13 (36–69)	153 ± 9 (141–163)	97 ± 6 (87–101)	9.3 ± 0.5 (8.8–10.0)

Behavior was a significant predictor of swimming speed, body length, stroke angle, and body angle, indicating that these parameters differed between the two behaviors (Table S3). The burst swimming behavior exhibited faster mean swimming speeds (0.28 m s^−1^; Table 3) than takeoff (0.15 m s^−1^; Table 4). Animals that exhibited burst swimming also used larger mean stroke angles (108°) than those in takeoff (98°; Tables 3, 4). Finally, in the burst swimming behavior, animals swam with a nearly horizontal body angle (mean = 178°), whereas animals swam with a steeper body angle (mean = 163°) in the takeoff behavior (Tables 3, 4). Behavior was not a significant predictor of power stroke duration, recovery stroke duration, phase lag, or beat frequency. For a typical burst swimming bout, animals swam at body-based *Re* on the order of 10^4^, and a typical pleopod stroke operated on the order of 10^3^, thus operating in the inertial realm.

**Fig. 3:**
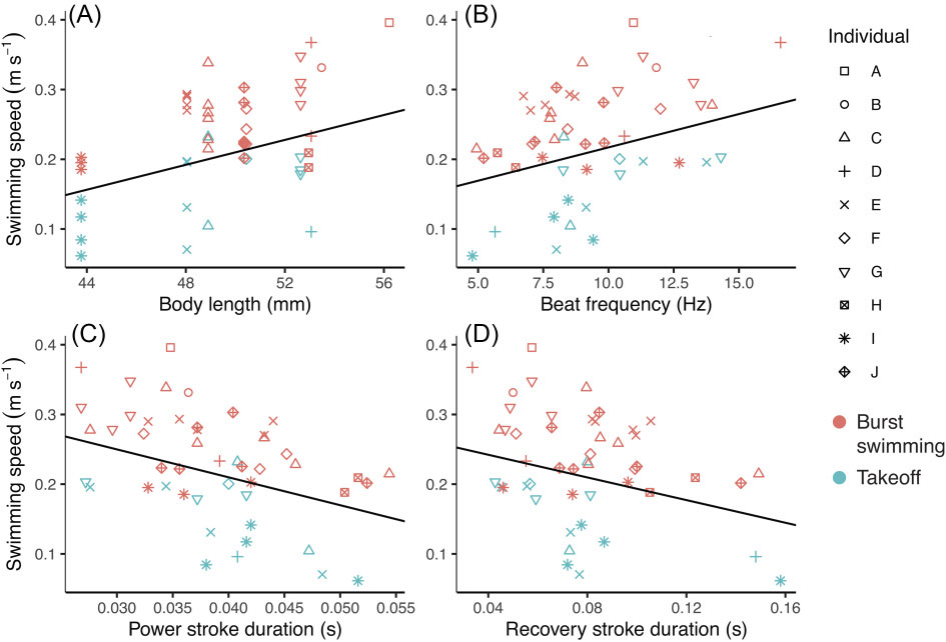
Swimming speed is best explained by behavior, body size, and temporal aspects of pleopod movement across the two behaviors. Statistically significant regression lines are plotted from the output of linear mixed models with ID and behavior included as random effects (see Methods; Table S5). Each symbol represents an individual and each data point represents one swimming bout. (A, B, C, D) Animals that exhibit the burst swimming behavior achieve faster swimming speeds (red symbols; mean = 0.28 m s^-1^) than in the takeoff behavior (blue symbols; mean = 0.15 m s^-1^). (A) Larger animals achieve faster swimming speeds. (B) More frequent pleopod strokes result in faster swimming speeds. (C, D) Both power stroke duration and recovery stroke duration are negatively correlated with swimming speed (i.e., the longer it takes the animal to complete a stroke, the slower their swimming speeds).

**Fig. 4:**
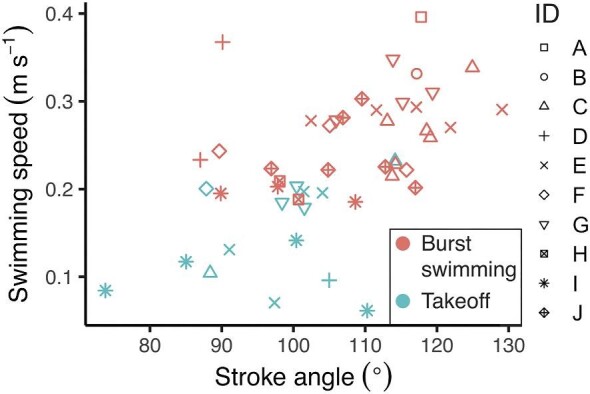
Stroke angle is associated with both swimming speed and behavior. When the behaviors are combined into a single analysis, stroke angle is positively correlated with swimming speed. When behavior is incorporated as a parameter in statistical models, stroke angle and swimming speed are not significantly correlated (see Tables S4, S5 and Methods). Each symbol represents an individual and each data point represents one swimming cycle.

Two sets of LMMs were used to determine which parameters best predict swimming speed. In the first set, we found that swimming speed was best predicted by behavior, body length, stroke angle, power stroke duration, beat frequency, and recovery stroke duration (in order of ascending ΔAIC score; Fig. 3, Table S4). When we included behavior as an additional random effect in the second set of LMMs, we found that swimming speed was still best predicted by power stroke duration, beat frequency, recovery stroke duration, and body length but no longer significantly predicted by stroke angle (Fig. 4, Table S5). In both sets of models, body angle and phase lag did not significantly predict swimming speed (Tables S4, S5).

**Fig. 5:**
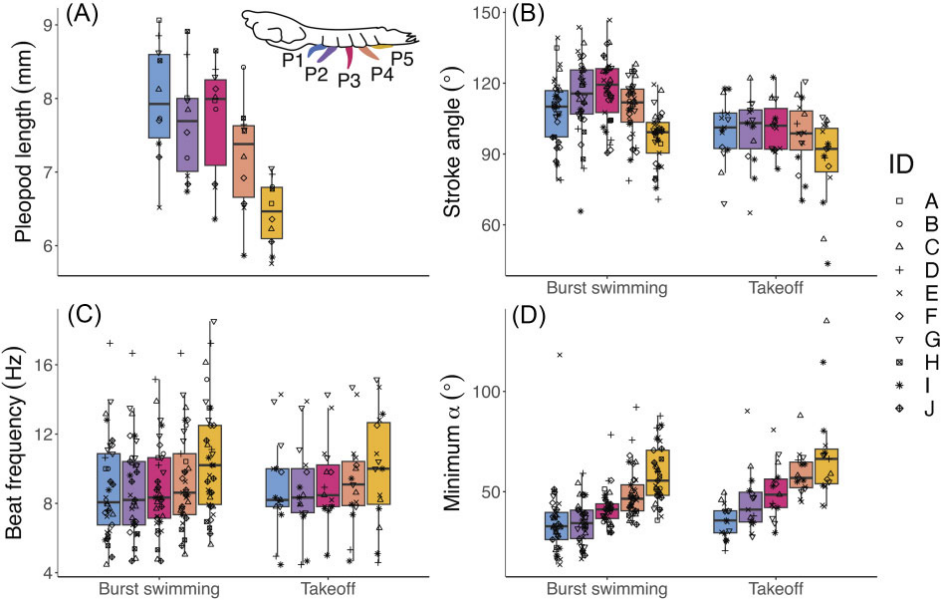
Stroke kinematics vary across the five pleopods. (A) The posterior-most pleopod (P5) is shortest and pleopod length generally increases anteriorly. (B) Stroke angle is greater and more variable across the five pleopods in the burst swimming behavior than during takeoff. In burst swimming, innermost pleopods (P2, P3) have larger stroke angles and P5 has the smallest stroke angle. (C) Beat frequency is largest in P5 across both behaviors. (D) Minimum α increases posteriorly, such that P1 starts the power stroke when closest to the abdomen and P5 starts the power stroke further away from the abdomen. (B, D) Given that minimum α increases posteriorly, stroke angle becomes oriented slightly posteriorly for P3, P4, and P5. Boxplot horizontal bars indicate the median value. Points are jittered horizontally to enhance visibility.

**Fig. 6:**
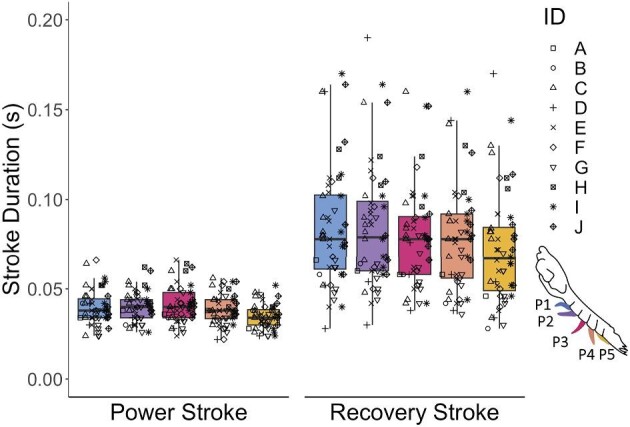
Stroke duration is highly consistent across pleopods during the power stroke compared to greater variability during the recovery stroke. Data are combined across both burst swimming and takeoff behaviors (see Results for statistical results showing no statistically significant difference between behaviors for stroke durations). Boxplot horizontal bars indicate the median value. Points are jittered horizontally to enhance visibility.

Stroke angle, minimum α, and maximum α statistically varied with the pleopod's position along the abdomen (Fig. 5, Table S6). Mean stroke angle was smallest in the posterior-most pleopod (P5) and largest in the innermost pleopod (P3) across both behaviors (Fig. 5B, Table 5). The inter-pleopod variation of stroke angle, or difference across the five pleopods, was greater in burst swimming than in takeoff (Fig. 5B). Minimum α increased posteriorly from an average of 35 to 59° in burst swimming and from 36 to 69° in takeoff (Fig. 5D, Table 5). Maximum α also increased posteriorly from an average of 147 to 161° in burst swimming and 141 to 163° in takeoff but reached its highest value in P4 and then decreased in P5 (Table 5). Both minimum α and maximum α exhibited a similar pattern of inter-pleopod variation across both behaviors. Pleopod length also statistically differed with pleopod position, such that P5 was shortest and P1 was longest (Fig. 5A, Tables 5, S6). Beat frequency significantly differed across the five pleopods and the highest value was exhibited by P5 (Fig. 5C, Tables 5, S6). Finally, pleopod position was also a significant predictor of power stroke duration but not recovery stroke duration (Table S6). Across both behaviors, power stroke duration maintained a narrow temporal range (3.0–5.1 s) compared to a more highly variable recovery stroke duration (4.4–14.8 s; Fig. 6).

In comparison with other species of mantis shrimp, *N. bredini* exhibited kinematics that were generally in the range of *O. scyllarus* but different than those of *O. havanensis* (Table 2). *N. bredini* and *O. havanensis* are comparable in body size and have similar ratios of appendage spacing (*G*) to appendage length (*L*) (*G/L* = 0.56), whereas *O. scyllarus* is twice as large as *N. bredini* and has a *G/L* = 0.34, meaning that the pleopods are more closely spaced (Table 2). *Odontodactylus havanensis* achieved a larger mean swimming speed of 24.1 BL s^−1^ (1.31 m s^−1^), larger mean stroke angle of 138°, and larger mean beat frequency of 16.8 Hz than *N. bredini* in this study (mean swimming speed: 5.40 BL s^−1^ or 0.28 m s^−1^, mean stroke angle: 108°, mean beat frequency: 9.8 Hz) (Campos et al. 2012; Table 2). On the other hand, *O. scyllarus* had a similar mean stroke angle (*O. scyllarus*: 110°, *N. bredini*: 108°), but exhibited a considerably lower mean swimming speed and mean beat frequency (2.6 BL s^−1^ and 4.2 Hz, respectively; Table 2; Garayev and Murphy 2021). Finally, mean phase lag ranged from 9.1 to 15% across the three compared species with no significant correlation between phase lag and swimming speed (Tables 2, S5).

**Fig. 7:**
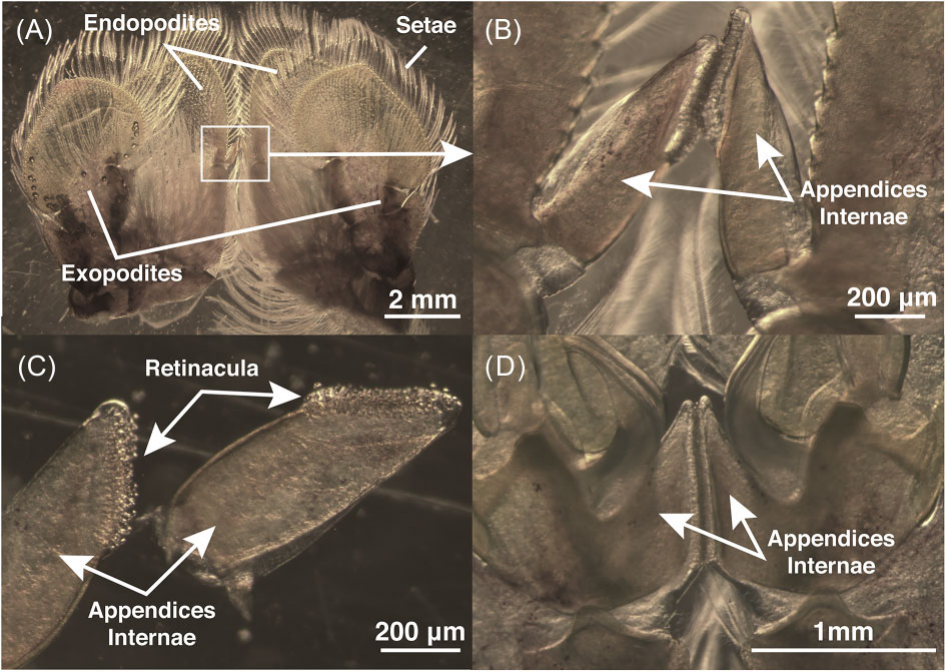
Pleopods, formed by paired exopodites and endopodites, are connected by appendices internae which are lined with tiny hooks (retinacula). (A) Anterior face of pleopod pair three (P3) with ventral toward the top of the page and abdominal attachment site toward the bottom of the page. (B) Zoomed in view of image in (A) shows the appendices internae which attach to each other with hook-like retinacula along their medial surfaces. (C) With the appendices internae cut and separated from pleopod pair four (P4), hook-like retinacula are visible. (D) More robust appendices internae are shown here on pleopod pair one (P1) compared to the less robust internae on pleopod pair three (P3) shown in (B). The appendices internae are more easily disconnected in the more posterior pleopod pairs.

In *N. bredini*, pleopods have connective structures linking the biramous lobes of the pleopod pairs together (Fig. 7). This connection is facilitated by small hook structures termed retinacula (plural; singular is retinaculum). Retinacula are located along the medial surfaces of the appendices internae and interlock with one another (Fig. 7). All five pleopod pairs have these connections. From posterior to anterior, the length of the appendix interna becomes greater and consequently the number of retinacula increases, such that more anteriorly positioned pleopods have more retinacula than posteriorly positioned pleopods.

## Discussion

Mantis shrimp (*N. bredini*) subtly modify coordination across their five pleopods, varying both spatial and temporal kinematics in tandem to achieve different behaviors and swimming speeds. Independent variation of the five pleopods reveals kinematic mechanisms for achieving forward propulsion while maintaining coordination. We identify several of these mechanisms, such as the inter-pleopod variation of stroke angle, posterior orientation of power strokes, and the use of kinematics to compensate for constraints. In bio-inspired robotic design of metachronal swimmers, stroke kinematics, such as stroke angle, are often fixed at a certain value across the five pleopods (Takagi 2015; Ford et al. 2021; Ford and Santhanakrishnan 2021a; Ford and Santhanakrishnan 2021b). However, a finer level of control may be possible with the incorporation of pleopod-specific kinematics in hybrid metachronal swimming. Our observation of appendices internae with varying robustness also offer a new window into potential passive and active dynamic pathways when using flexible paddles to swim. We critically assess these findings below and conclude by comparing our findings with previously published data from other mantis shrimp species that also exhibit hybrid metachronal swimming.

Takeoff and burst swimming behaviors are distinguished by different swimming speeds, stroke angles, and body angles (Figs 3, 4, Table S3). Specifically, burst swimming behavior is characterized by faster swimming speeds, larger stroke angles, and nearly horizontal body angles, whereas the takeoff behavior is characterized by slower swimming speeds, smaller stroke angles, and steeper body angles (Tables 3, 4). It should be noted that body length slightly confounded our findings because the burst swimming trials were comprised of slightly larger individuals (Table 3) than those in the takeoff trials (Table 4). Previous studies define and compare distinct swimming modes via similar parameters in other metachronal swimmers (Murphy et al. 2011; Ruszczyk et al. 2022). The two behaviors in this study represent ecologically relevant locomotor modes used by mantis shrimp during navigation of benthic marine environments. Burst swimming represents quick bursts or a darting behavior used to pursue prey or avoid predation. Takeoff represents the transition from substrate to open water or even the upward motion required to ambush evasive prey, as seen in some spearing mantis shrimp species.

Coordination among closely spaced pleopods must be maintained during any modification to stroke kinematics. Robotic modeling suggests that larger stroke angles within a hybrid metachronal system result in higher swimming speeds by generating stronger wake flows (Ford et al. 2021). Our similar finding that larger stroke angles are positively associated with swimming speed across behaviors (Fig. 4, Table S4) now raises a subsequent question: How do mantis shrimp effectively achieve larger stroke angles (consequently generating greater thrust) without interference of neighboring pleopod strokes? We find that, instead of increasing stroke angle uniformly across each pleopod, mantis shrimp vary stroke angle across the five pleopods, such that innermost pleopods achieve the largest stroke angles compared to outer ones (Fig. 5B). Furthermore, a greater variation of stroke angle across pleopods is used at faster speeds as compared to little variation across pleopods in the takeoff behavior (Fig. 5B). This strategy allows pleopods to generate higher thrust via larger strokes, while avoiding collision with neighboring appendages.

A similar inter-pleopod pattern of stroke angle is found in another mantis shrimp species, *O. havanensis*, which also has a comparable appendage spacing to appendage length ratio (*G/L*) to *N. bredini* (Campos et al. 2012; unpublished data extracted by Ruszczyk et al. 2022). In both *O. havanensis* and *N. bredini*, stroke angle is smallest in P5, then increases anteriorly to the largest value in P3, and finally decreases anteriorly to the second-smallest value in P1 (Fig. 5B). This pattern of variation among pleopods remains similar between the two species despite different average stroke angles (138° in *O. havanensis*, 108° in *N. bredini*). It is possible that other hybrid metachronal swimmers or animals with similar *G/L* appendage spacing use a kinematically-similar strategy of inter-pleopod variation to achieve larger stroke angles and therefore greater thrust, although Garayev and Murphy (2021) report that stroke angle increases with more anterior pleopods in *O. scyllarus* (*G/L *= 0.34).

Inter-pleopod stroke angle patterns differ considerably in comparisons between hybrid and complete metachrony. Complete metachronal swimmers, such as krill (*Euphausia superba*) and mysid shrimp (*Americamysis bahia*), exhibit the largest stroke angle in P5 that then decreases posteriorly to the smallest stroke angle in P1 (Murphy et al. 2011; Ruszczyk et al. 2021). Yet, mantis shrimp in this study exhibit the smallest stroke angle in P5. These differences may be explained by the need to transition (or not) between a near-synchronous recovery stroke and metachronal power stroke. In hybrid metachronal swimming, P5 serves as the first swimming appendage to initiate the power stroke, thereby constraining the pleopod's motion to a smaller stroke angle possibly given the need to quickly transition between these two phases. For both hybrid and complete metachronal swimmers, inter-pleopod variation of stroke angle encourages future investigations regarding comparative kinematics and morphology across different coordination strategies and scales.

Not only does the independent variation of pleopod kinematics allow for modifiable coordination strategies (such as with a larger stroke angle in the innermost pleopod), but it also provides the ability to compensate for constraints across pleopods. For instance, P5 is the shortest pleopod and exhibits the smallest stroke angle—possibly related to its role in initiating the power stroke and quickly transitioning between recovery-power phases. Along with these constraints, P5 exhibits a higher mean beat frequency than other pleopods (Fig. 5C, Table 5). This suggests that faster strokes may compensate for a smaller stroke angle and shorter paddle length. Therefore, independent modification of kinematics across pleopods provides unique pathways for achieving propulsion and coordination suited to specific constraints.

Statistical analyses indicate that temporal parameters and body size are better predictors of swimming speed than stroke angle; however, stroke angle, although confounded with behavior, likely also plays a substantial role in swimming speed (Figs 3, 4). Other studies of metachronal swimmers show that higher beat frequencies (a temporal parameter; Kils 1981; Alexander 1988; Murphy et al. 2011; Heimbichner Goebel et al. 2020; Ruszczyk et al. 2021; Ruszczyk et al. 2022) and larger stroke angles (a spatial parameter; Murphy et al. 2011; Ford et al. 2021) can both contribute to faster swimming speeds. We found that power stroke duration, recovery stroke duration, and beat frequency statistically predict swimming speed (Fig. 3, Tables S4, S5). Each of these parameters are functions of time, and modifying one temporal variable requires modification of another. For example, a faster power stroke duration inherently results in a higher beat frequency. However, while we found that stroke angle is a significant predictor of swimming speed across behaviors (Table S4), it is not a significant predictor of swimming speed when behavior is included as a random effect in the linear mixed models (Fig. 4, Table S5). In other words, stroke angle differentiates the two behaviors and contributes to the difference in mean swimming speeds between the two behaviors (Tables 3, 4). Larger stroke angles contribute to faster swimming speeds, but with the caveat that this relationship is confounded by the animal's behavior. Therefore, we conclude that stroke angle is an important kinematic between behaviors as well as across swimming speeds.

While studies of complete metachrony show that swimming speed increases with phase lag (Ford and Santhanakrishnan 2021a; [Bibr bib16][Bibr bib16]), we did not find a significant relationship between phase lag and swimming speed in mantis shrimp. Mean phase lag varies slightly across the four consecutive pairs but remains within a constrained range across both behaviors (8.9–9.9%; Table S7). Robotic modeling of hybrid metachrony indicates that a phase lag of 10% results in the highest swimming speed for a large range of stroke angle values (Ford et al. 2021). Our biological data is consistent with this finding, with a phase lag of roughly 9.3% across both behaviors (Table S7). Unlike stroke angle, we did not observe much variation in phase lag among pleopods (Fig. S2) nor an overall correlation between stroke angle and phase lag—supporting the finding that stroke angle is not limited by phase lag in hybrid metachronal systems (as suggested by Ford et al. 2021).

Analyzing combinations of stroke kinematics provides insight into other possible mechanisms that allow mantis shrimp to achieve faster swimming speeds. For instance, the direction of thrust generated during power strokes is represented by the combination of α and stroke angle. α represents the pleopod's position in relation to the animal's body, where minimum α occurs when the pleopod is positioned for the start of the power stroke (pointing anteriorly) and maximum α occurs when the pleopod is tucked closest toward the body and is about to start the recovery stroke (pointing posteriorly; Fig. 2). In effect, increasing minimum and maximum α values posteriorly across the five pleopods (Fig. 5D, Table 5) causes stroke angle to be oriented downward for P1 and P2 and slightly posteriorly for P3, P4, and P5. This orientation indicates that posterior pleopods generate more forward thrust and anterior pleopods generate more upward thrust. In complete metachronal swimmers, a posteriorly moving jet at large speeds results in forward motion (Yen et al. 2003; Catton et al. 2011). Although hybrid metachronal swimmers generate a more dispersed wake compared to narrower jets produced by complete metachronal swimmers (Ford et al. 2021), the backward orientation of power strokes may similarly contribute to greater forward propulsion, as seen in the burst swimming behavior.

Furthermore, kinematics and paddle size are dynamically modified between power and recovery stroke phases. Like other metachronal swimmers, mantis shrimp exhibit a faster power stroke compared to the recovery stroke (also referred to as temporal asymmetry; Fig. 6; Kiørboe et al. 2010; Herrera-Amaya et al. 2021). In addition, the surface area of the pleopod is dynamically modulated between power-recovery phases, which is common in other metachronal swimmers (Lenz et al. 2004; Murphy et al. 2011; Campos et al. 2012; Lenz et al. 2015). During the power stroke, we observed lateral extension of distal segments of the pleopod to effectively increase the paddle's surface area and generate thrust normal to the direction of the animal's motion. During the recovery stroke, the distal segments then decrease their angle relative to each other and the pleopod folds posteriorly to decrease surface area and minimize drag (Supplementary Videos S1, S2). Setae also aid in maximizing and minimizing surface area by splaying outward during the power stroke and folding posteriorly during the recovery stroke. Therefore, net fluid displacement and dynamic paddle surface areas are likely important to consider in tandem with and beyond stroke kinematics presented here.

Across mantis shrimp species, scaling of kinematics and swimming speed is not immediately evident (Table 2; Ruszczyk et al. 2022). Instead, we found that unique combinations of kinematics can result in either similar or varying swimming speeds across species. *Odontodactylus havanensis* achieved considerably faster swimming speeds (21.0–28.7 BL s^−1^) with a higher beat frequency (16.8 Hz) and larger stroke angle (124–154°) than our similarly sized *N. bredini* (Campos et al. 2012, Ruszczyk et al. 2022). On the other hand, *O. scyllarus*, which is twice the size as *N. bredini*, were reported to achieve slower speeds (1.8–3.4 BL s^−1^) with slower beat frequencies (3.6–4.8 Hz), larger phase lags (14.5–15.5%), and similar stroke angles (106–114°), although *O. scyllarus* were likely not performing at maximal speeds. A previous study of *N. bredini* locomotion found a larger stroke angle (117°), larger phase lag (14.8%), and lower beat frequency (8.5 Hz) than we measured for the same species, yet the animals achieved a similar swimming speed as the ones in this study (Ford et al. 2021). Therefore, different kinematic combinations can be used to achieve similar swimming speeds.

### Pleopod appendices internae and retinacula morphology

Mantis shrimp pleopod pairs are connected with appendices internae, which are lined with hook-like structures termed retinacula (Fig. 7). These connections are likely an important and under-recognized aspect of metachronal locomotion. Retinacula (also referred to as cincinnuli in other literature) have been found across many orders of crustaceans (Grams and Richter 2021). Appendices internae are cuticular protrusions from the endopodites, which have been proposed to serve as an adhesive, coupling mechanism to enforce synchronism of left and right pleopods during propulsion (Bouchon and Chaigneau 1991; Ahyong and Harling 2000). They have also been proposed to function in copulatory processes (Bauer 1976).

In mantis shrimp, the appendices internae connect most strongly in the anterior-most pleopod (P1) and decrease in robustness posteriorly such that the appendices internae of the posterior pleopods detach from each other more easily (Fig. 7). Anterior pleopods have larger appendices internae and thus more retinacula lined along the medial surface (Fig. 7D). Easier detachment of the posterior pleopods may allow increased lateral range of movement and thus maneuverability (see Fig. S3). Stronger attachment in anterior pleopods may be needed given greater force exerted in the perpendicular plane or the strength of vortices shed during the propulsive stroke (see Garayev and Murphy 2021).

In other metachronal swimmers, retinacula aid in coordinating pleopod pairs and allow for lateral flexibility (Nishida et al. 2013). This lateral flexibility increases the range of angles between the endopodites and exopodites on either side of the pleopod pair, thereby enhancing maneuverability. During the recovery stroke, pleopods are often retracted toward the centerline of the body, decreasing their surface area (and thus drag) as they move anteriorly to prepare for the power stroke (Murphy et al. 2011). The structure and orientation of the appendices internae coupled with potential recoil or adhesive forces of the retinacula can serve as a passive mechanism in transition into the recovery stroke where less or little energy is required for pleopod retraction. This has also been proposed for a similar structure (fused setules) in barnacle larvae (Lamont and Emlet 2018). Future studies are needed to determine the strength of retinacula and the function of detaching pleopod pairs in mantis shrimp locomotion.

A staggering diversity of organisms use various forms of metachrony. Our study reveals how mantis shrimp modify stroke kinematics, while maintaining pleopod coordination to accomplish varying swimming behaviors. Future investigations with strong biological experimental datasets will likely reveal new principles of control, morphology-fluids integration, and scaling across this highly effective form of locomotion.

## Supplementary Material

obad019_Supplemental_Files

## Data Availability

Data and code are freely available in the Dryad Digital Repository (Hanson et al. 2023): https://doi.org/10.5061/dryad.x95x69pq6.
