# Supplementary material for: Mantis Shrimp Locomotion: Coordination and Variation of Hybrid Metachronal Swimming
Source: Integr Org Biol. 2023 Jun 27;5(1):obad019. doi: 10.1093/iob/obad019 (PMC10303694; doi:10.1093/iob/obad019)
Supplement: obad019_Supplemental_Files [file obad019_supplemental_files.zip › Revision_SupplementaryMaterials_050123v2.docx]

# Supplementary Materials

**Table S1. Comparison of two methods for calculating stroke angle across individuals in each behavior.** There were minor differences between calculating stroke angle using the four-point digitization method (modified from Kagaya and Patek (2016)) and by subtracting minimum α from maximum α values (α subtraction method) (see Methods). Across both methods, stroke angle values were mostly within 1°–4° of each other. Stroke angle was averaged across 33 cycles in the burst swimming behavior and across 15 cycles in the takeoff behavior. Values are reported as means and ranges. Bottom row contains the overall mean ± standard deviation of means followed by the range in parentheses. The α subtraction method requires the long axis of the body to be digitized (Fig. S1), which is also useful for approximating body angle and α angles. On the other hand, the four-point digitization method reduces digitizing error by not requiring the long axis of the body to be digitized. Therefore, we report values of stroke angle from the four-point digitization method in this study and α angle from digitizing the long axis of the body.


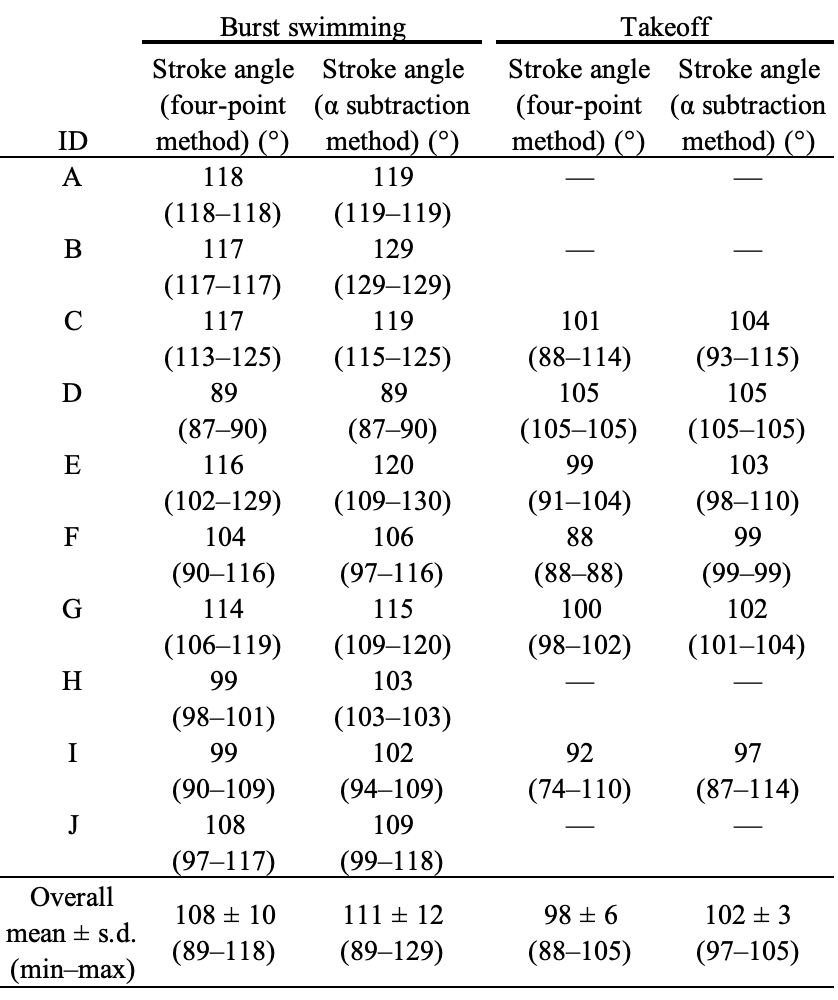


# **Table S2. Comparison of two methods for calculating stroke angle by each pleopod.** There were minor differences between the four-point digitization method (modified from Kagaya and Patek (2016)) and subtracting minimum α from maximum α values (α subtraction method; see Methods). Stroke angle was averaged across 33 cycles in the burst swimming behavior and across 15 cycles in the takeoff behavior. Values are reported as means and ranges. Bottom row contains the overall mean ± standard deviation of means followed by the range in parentheses. In this study, we report values of stroke angle from the four-point digitization method and α angle from digitizing the long axis of the body.


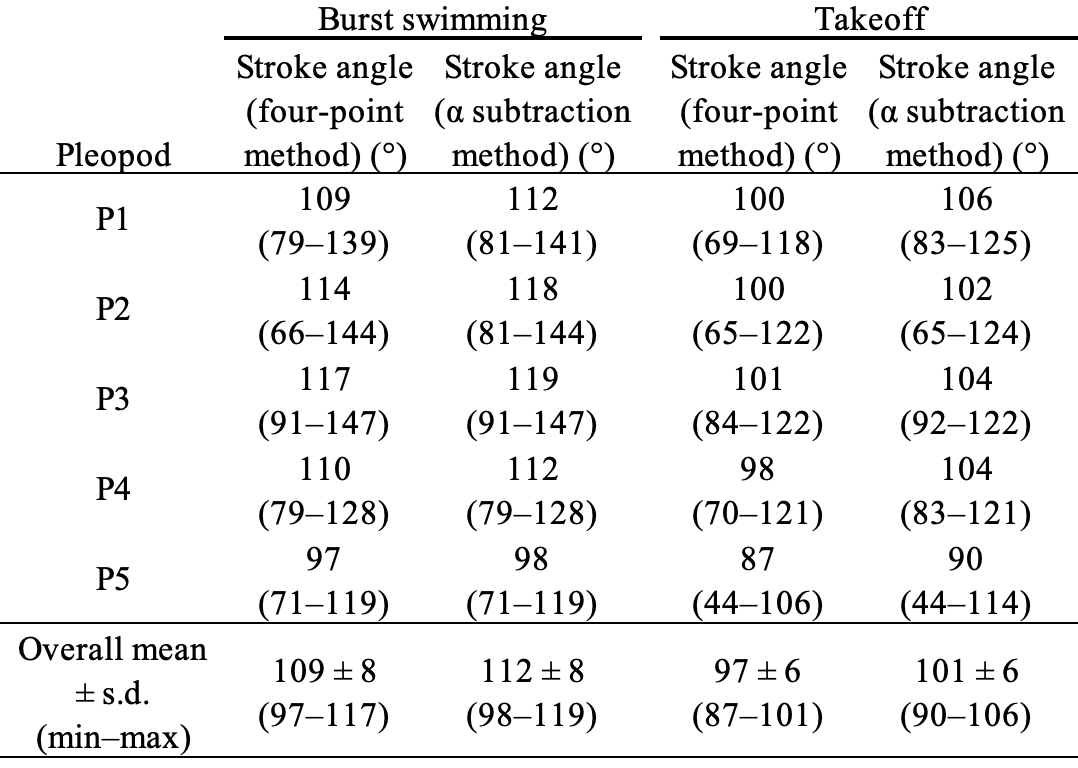


# **Table S3. ΔAIC scores output from linear mixed models (LMM) indicate the statistical effect of behavior on swimming parameters.** See Statistical Analyses section in the Methods for details.


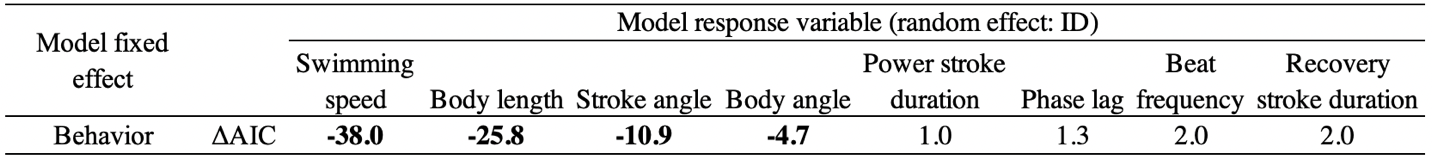


#
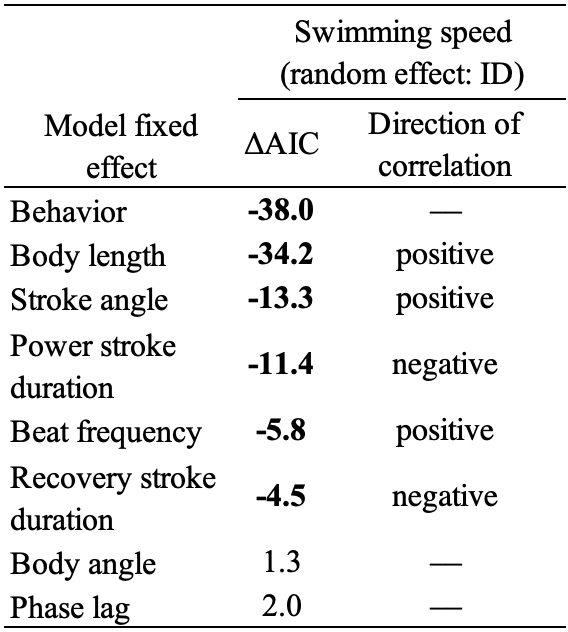
**Table S4. ΔAIC scores output from linear mixed models (LMM) indicate which parameters significantly explain variation in swimming speed.** See Statistical Analyses section in the Methods for details.

#
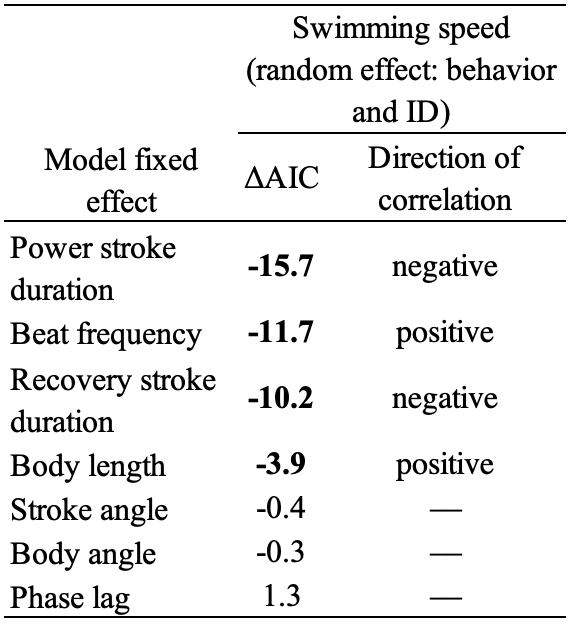
**Table S5. ΔAIC scores output from linear mixed models (LMM) indicate which parameters significantly explain variation in swimming speed when behavior is included as a random effect.** See Statistical Analyses section in the Methods for details.

**Table S6. ΔAIC scores output from linear mixed models (LMM) indicate the statistical effect of pleopod position (P1–P5) on stroke parameters.** See Statistical Analyses section in the Methods for details.

**
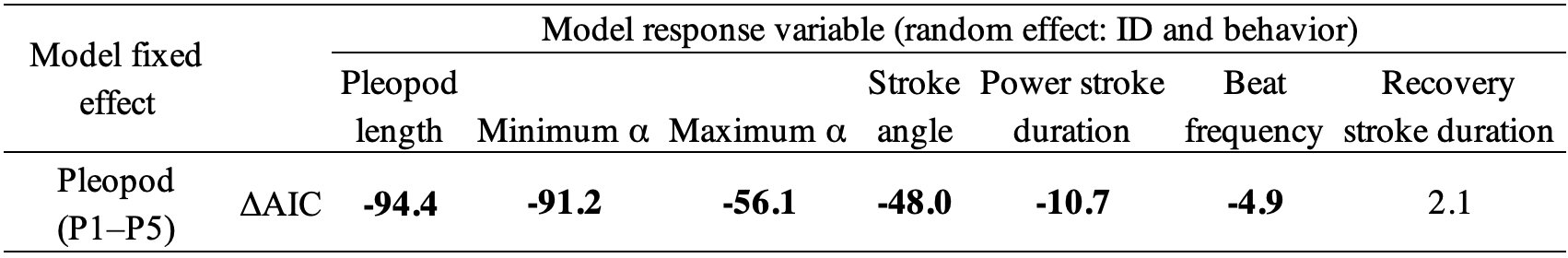
**

**Table S7. Phase lag across consecutive pleopod power strokes (P5–P4, P4–P3, P3–P2, P2–P1).** Phase lag was averaged across 33 cycles in the burst swimming behavior and across 15 cycles in the takeoff behavior. Values are reported as means and ranges. Bottom row contains overall mean ± standard deviation of means followed by the range in parentheses. Overall phase lag is reported as an average of both behaviors.


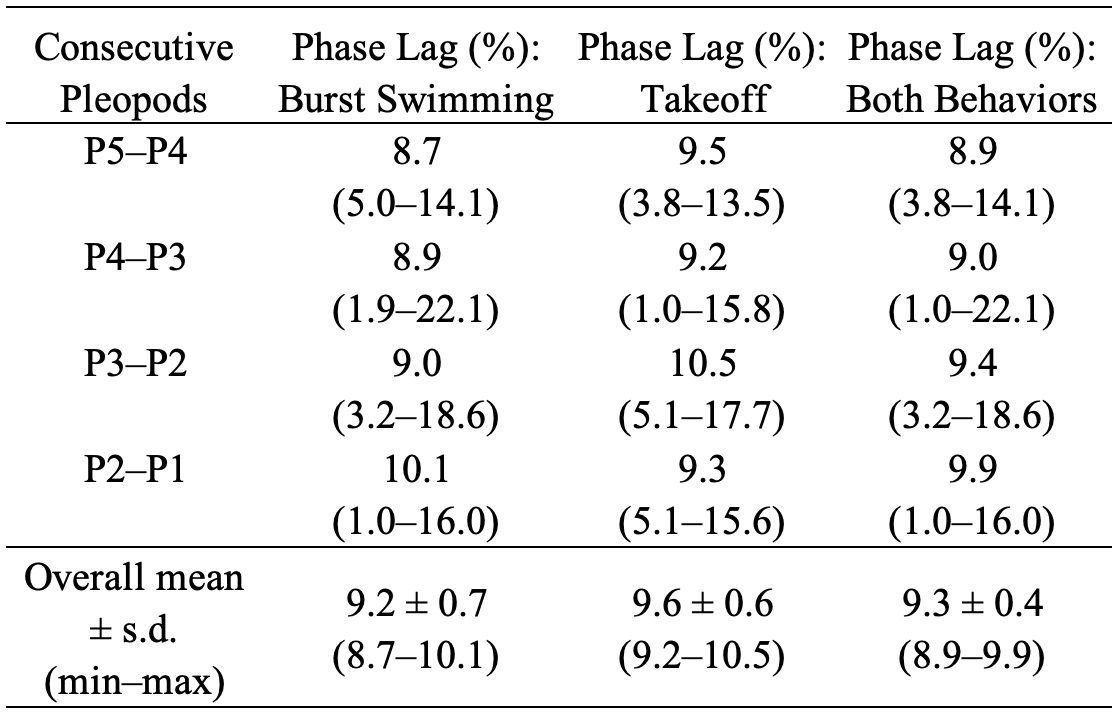


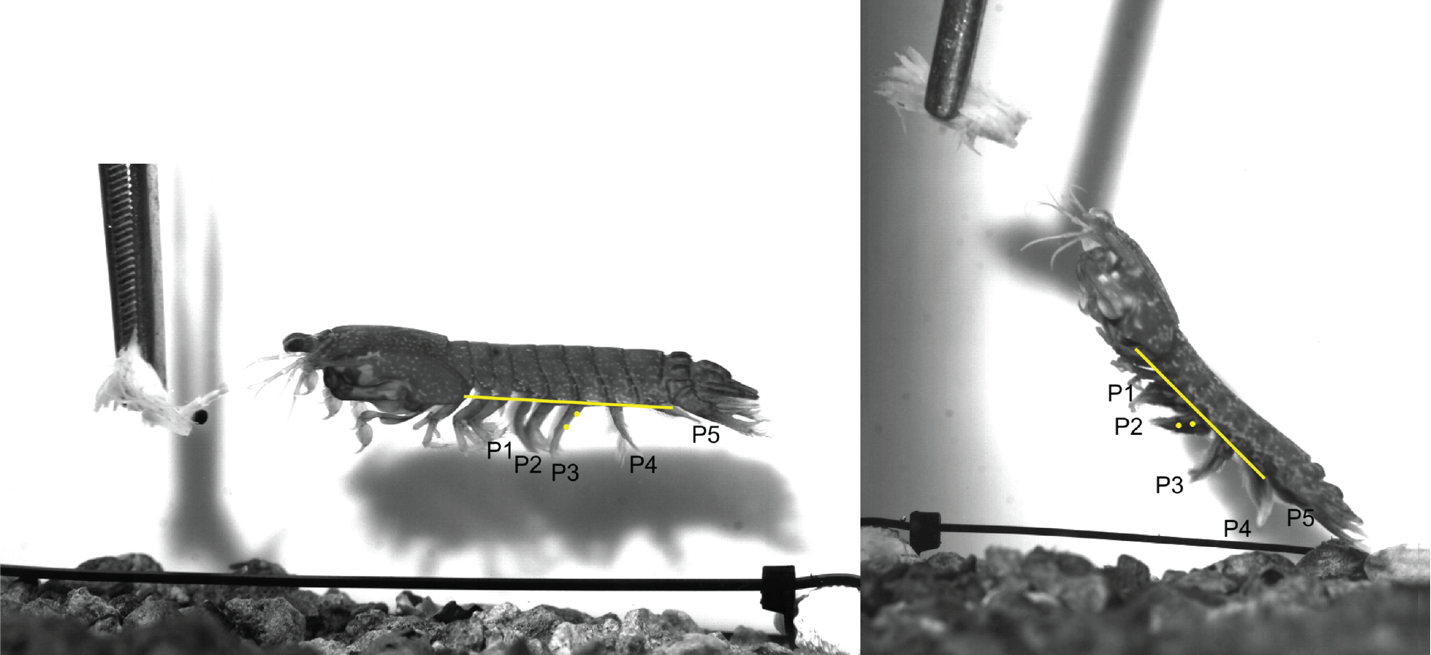


**Figure S1.** **Digitization of the long axis of the body.** To calculate body angle and appendage angle (α), we digitized a line along the body, representing the long axis of the body for the abdominal segment associated with the pleopod being digitized (P3 in A; P2 in B). The line was digitized at the start of each pleopod’s power stroke from the high speed videos. The yellow points represent the two digitized points placed on the pleopod’s joints from the four-point method (see Methods), also used to calculate stroke angle. Body angle is expressed as an angle relative to the substrate (or positive x-axis) for left-facing animals.

#


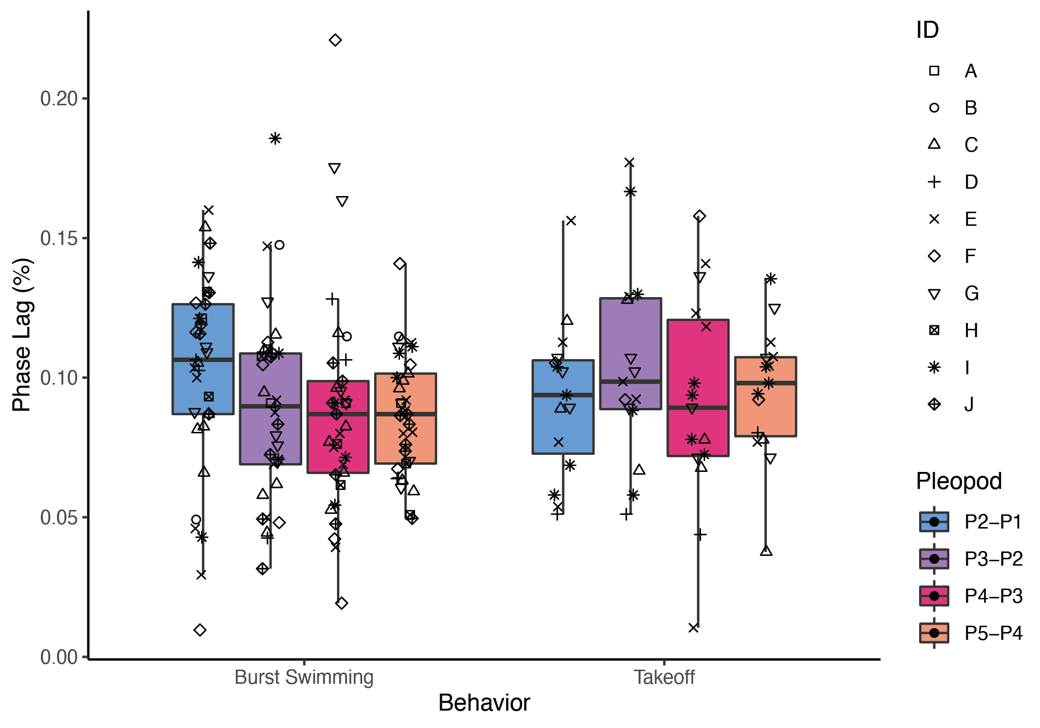


**Figure S2. Median phase lag is fairly consistent across consecutive pleopod strokes.** Boxplot horizontal bars indicate the median value.

# **
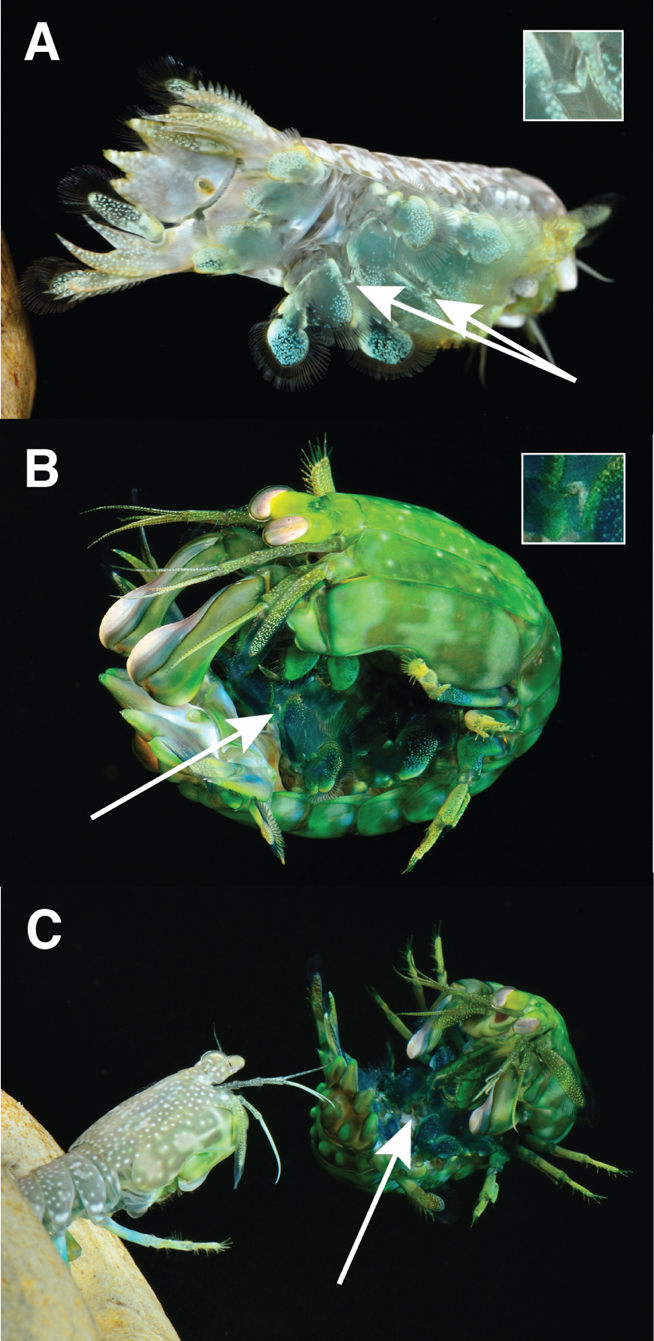
Figure S3. Appendices internae connect pleopod pairs (arrows).** Retinacula, connective micro-hook structures, line the adjoining surfaces of the appendices internae. **(A)** A ventral, posterior view of a female *N. oerstedii* swimming (into the page). **(B)** A male *N. oerstedii* forms a defensive posture by coiling up the telson, such that the pleopod connections are visible. **(C)** A female (left) and male (right) interact at the entrance to a burrow. Pictures reproduced with permission from Roy Caldwell.

**Supplemental Videos**

**Video S1. A typical example of burst swimming behavior.** One complete swimming cycle was digitized (from the start of the posterior-most pleopod’s recovery stroke to the end of the anterior-most pleopod’s power stroke). The body length of the animal in this video is 52.63 mm. Original filming rate: 500 fps. Playback speed: 30 fps.

**Video S2. A typical example of takeoff behavior.** While the entire swimming movement in this video represents the takeoff behavior, we digitized the first swimming cycle when the animal’s appendages were not touching the substrate (from 0.0201 seconds to 0.0407 seconds; see Methods). Body length is 48.04 mm. Original filming rate: 500 fps. Playback speed: 30 fps.
